# Preliminary Investigation on Auto-Thermal Extrusion of Ground Tire Rubber

**DOI:** 10.3390/ma12132090

**Published:** 2019-06-28

**Authors:** Łukasz Zedler, Daria Kowalkowska-Zedler, Henri Vahabi, Mohammad Reza Saeb, Xavier Colom, Javier Cañavate, Shifeng Wang, Krzysztof Formela

**Affiliations:** 1 Department of Polymer Technology, Faculty of Chemistry, Gdańsk University of Technology, Gabriela Narutowicza 11/12, 80–233 Gdańsk, Poland; 2 Department of Inorganic Chemistry, Faculty of Chemistry, Gdańsk University of Technology, Gabriela Narutowicza 11/12, 80–233 Gdańsk, Poland; 3 Université de Lorraine, CentraleSupélec, LMOPS, F-57000 Metz, France; 4 Laboratoire Matériaux Optiques, Photoniques et Systèmes, CentraleSupélec, Université Paris-Saclay, 57070 Metz, France; 5 Department of Resin and Additives, Institute for Color Science and Technology, 16765-654 Teheran, Iran; 6 Department of Chemical Engineering, Universitat Politècnica de Catalunya Barcelona Tech, Carrer de Colom, 1, 08222 Terrassa, Barcelona, Spain; 7 Department of Polymer Science and Engineering, Shanghai Jiao Tong University, Shanghai 200240, China

**Keywords:** waste tires, recycling, reclaiming/devulcanization, extrusion, auto-thermal extrusion, reclaimed rubber, structure-property relationships

## Abstract

Ground tire rubber (GTR) was processed using an auto-thermal extrusion as a prerequisite to green reclaiming of waste rubbers. The reclaimed GTR underwent a series of tests: thermogravimetric analysis combined with Fourier-transform infrared spectroscopy (TGA-FTIR), scanning electron microscopy (SEM), Fourier-transform infrared spectroscopy (FTIR), and static headspace and gas chromatography-mass spectrometry (SHS-GC-MS) in order to evaluate the impact of barrel heating conditions (with/without external barrel heating) on the reclaiming process of GTR. Moreover, samples were cured to assess the impact of reclaiming heating conditions on curing characteristics and physico-mechanical properties. Detailed analysis of the results indicated that the application of auto-thermal extrusion is a promising approach for the sustainable development of reclaiming technologies.

## 1. Introduction

The ever-increasing development of the global automotive market contributes to the increase in the demand for car tires, and thus the number of waste tires increases. Annually, approximately 1000 million tires are not suitable for further use or retread and according to predictions, this number will increase approximately to 1200 million pieces by 2030 [1]. The most common methods of waste rubber management are dispsal and combustion; the former is forbidden nowadays and the second might increase the production of greenhouse gases (CO_x_ and NO_x_) as well as toxic substances [2]. The legislation on the management of waste rubbers introduced by the European Union in 1999 [3,4] resulted in increased interest in the development of new methods for the utilization of waste rubbers. A promising approach to the material recycling of waste tires is reclaiming [5,6,7]. Reclaiming of ground tire rubber (GTR) (sometimes called “devulcanization” in the literature) consists in the destruction of the cross-linked structure involving cross-link scission and degradation of polymeric chains. The obtained products (reclaimed GTR) should be characterized by better processing properties than GTR, allowing easier shaping and further vulcanization. Due to the changes in the physical and chemical structure of GTR, the obtained reclaimed rubbers can be used in the manufacturing of new rubber compounds.

Reclaiming of GTR can be obtained by thermal, mechanical, chemical, and biological methods or their combinations, which have recently been described in the literature [8,9,10]. It should be pointed out that recent studies in this field are usually focused on combining low temperatures and short processing times. For example, Ghosh et al. [11] carried out low-temperature devulcanization of styrene-butadiene rubber (SBR) vulcanizate using bis(3-triethoxysilyl propyl)tetra-sulfide (TESPT) as a devulcanizing agent. Grounded SBR was preliminarily soaked with various amounts of TESPT for 24 h and subsequently transferred to open two-rolls, where it was milled at temperatures between 65 and 70 °C. Obtained reclaimed rubbers were mixed with a curing system and vulcanized according to the curing characteristics. The authors indicated that it is possible to obtain a revulcanized product with superior mechanical and thermal properties using the aforementioned process conditions.

Cheng et al. [12] carried out reclaiming of GTR with aromatic oil at a relatively low temperature. During the process, three different temperatures of 140, 160, and 180 °C were applied. The results showed that the process can be successfully implemented at low temperatures (140 °C) with a high sol fraction content (68.3 wt.%). The reclaiming process of GTR allows for a product with a high sol content to be obtained, which could be used as a plasticizer in rubber compounding and as a modifier in road pavements.

Saiwari et al. [13] reported that the temperature is the most important parameter influencing the efficiency of thermo-mechanical reclaiming of different unfilled elastomers (natural rubber, styrene-butadiene rubber, butadiene rubber, and chlorinated butyl rubber). This confirms that the GTR reclaiming process should be performed at a reasonably low temperature. From an industrial point of view, however, the most crucial parameters to be considered in devulcanization/reclaiming methods would be met by extrusion technologies. This approach allows for continuity and short time, high mixing efficiency, high capacity, and good quality of the obtained products. Moreover, the versatility and diversity of extrusion lines are also a huge advantage for rubber recycling technologies. 

Nowadays, reactive extrusion of waste rubbers in twin screw extruders is a relatively well-known method of rubber recycling, which was invented two decades ago by Toyota R&D Division [14]. Although this technology was successfully applied for industrial scale reclaiming of ethylene-propylene-diene monomer rubber, the serious problem is still the reclaiming of GTR [15]. This is due to the presence of styrene-butadiene rubber in waste tires, which has a tendency to secondary cross-linking caused by oxygen and high temperatures. Another issue is the complex composition of waste tires, which can affect process reproducibility and the quality of the obtained products. Moreover, the extrusion parameters involved in the reactive processing must be properly chosen, otherwise the uncontrolled exothermic oxidation in waste rubber may occur, causing degradation of the polymeric chains and the emission of a high amount of odorous and toxic gases [16,17]. In order to reduce or eliminate this technological issue, a relatively low operating temperature during devulcanization/reclaiming via extrusion or high shear thermo-kinetic mixing seems to be a very promising route [18,19,20]. The literature indicates that low temperature reclaiming of GTR allows for selective scission of cross-linked bonds instead of the main chain scission, directly affecting the mechanical properties of the final product. Moreover, a low temperature affects the intensification of shear forces, limiting secondary cross-linking and reducing energy cost and the amount of emitted gases.

Recently, Seghar et al. [21] studied the effect of the extruder barrel temperature (in the range of 80–220 °C) on the thermo-mechanical devulcanization efficiency of post-production waste natural rubber (waste obtained by injection molding). The obtained results indicate the self-heating phenomenon of waste rubber during extrusion in the whole studied range of temperatures. However, this effect was more visible in the lower temperature range (80–140 °C). This phenomenon is related to the combined effect of two main factors. The first factor is high shear forces acting on the material, which are transferred into heat during extrusion. The second factor is related to exothermic reactions, which occur during reclaiming/devulcanization of GTR. To sum up, the self-heating phenomenon allows a reduction of the energy consumption during rubber recycling [22], which has a beneficial effect on the economic and sustainable development of waste tire recycling technologies.

In this paper, preliminary studies on the auto-thermal reclaiming of GTR in a co-rotating twin-screw extruder are presented. Two different heating conditions were applied: (i) constant barrel temperature at 60 °C and (ii) auto-thermal (without external heating of extruder barrel). Obtained samples were conditioned for at least 24 h. Subsequently, reclaimed GTR was mixed with a sulfur curing system and then vulcanized. 

For a better understanding of the changes in the physical and chemical structure of GTR, thermal analysis of GTR samples (reclaimed by the auto-thermal method), taken from different parts of a extruder barrel, was conducted. Static headspace and gas chromatography-mass spectrometry was used to compare the differences in the type and amount of volatile organic compounds (VOCs) generated from GTR samples. Moreover, the influence of the reclaiming method on the curing characteristics and physico-mechanical properties of cured GTR was investigated.

## 2. Materials and Methods 

### 2.1. Materials

**Figure 1:**
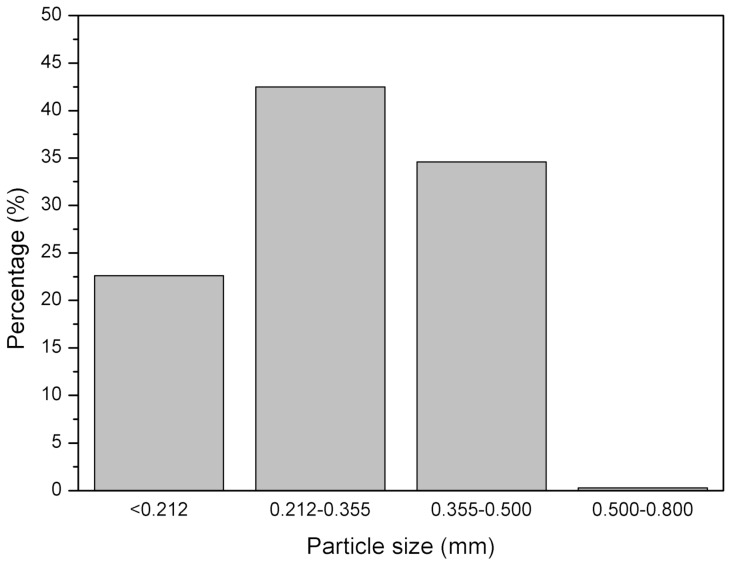
GTR particle size distribution.

Ground tire rubber (GTR) was received from Grupa Recykl S.A. (Śrem, Poland). GTR was obtained by ambient grinding of used tires (a mix of passenger car tires and truck tires). The particle size distribution of the used GTR is presented in Figure 1. Curing additives of a commercial grade were supplied by Standard Sp. z o.o. (Lublin, Poland).

### 2.2. Reclaiming of GTR

The reclaiming of GTR was carried out with the continuous method using a co-rotating twin-screw extruder EHP 2 × 20 from Zamak Mercator Sp. z o.o. (Skawina, Poland). The extruder has 11 heating/cooling zones with a screw diameter of 20 mm and an L/d ratio of 40. Detailed information about the screw configuration was presented in our previous work [18]. Ground tire rubber was added into the hopper at constant feed rate using a gravimetric feeder from Hydrapress Sp. z o.o. (Białe-Błota, Poland).

**Figure 2:**
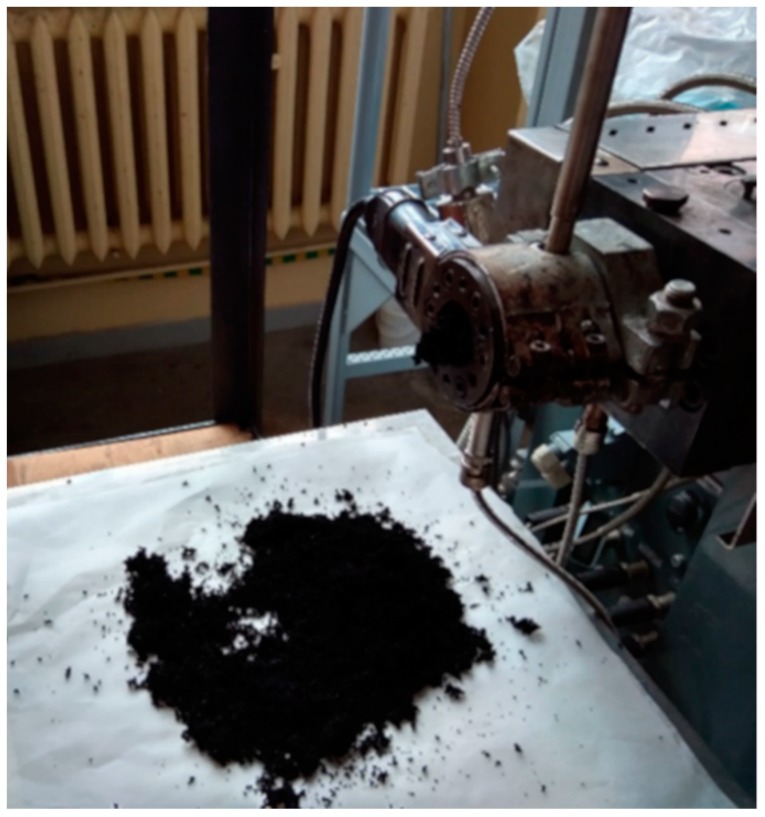
The appearance of GTR directly after auto-thermal reclaiming.

A summary of the extrusion conditions during the reclaiming of GTR is shown in Table 1. Two types of heating methods were applied: (i) Constant barrel temperature at 60 °C (coded as GTR-EXT) and (ii) auto-thermal (with disabled barrel heaters) (coded as GTR-AUTO EXT). In the first case, all the heating zones were set to 60 °C and the process proceeded with a constant heat supply. In the second solution, the heating zones were also heated to 60 °C, however, as soon as the process had reached stability, the heating/cooling system of the barrel was turned off. The auto-thermal conditions during reclaiming were set up as described in the patent [23]. The process was carried out on a laboratory scale with a capacity of 2.5 kg/h. It should be pointed out that the screw torque values for both heating methods were similar, which indicates that auto-thermal extrusion allows a reduction of the energy used during GTR reclaiming. The appearance of GTR after auto-thermal reclaiming is presented in Figure 2.

### 2.3. Curing of Reclaimed GTR

Reclaimed GTR obtained by the procedure described above was mixed with a sulfur curing system and processed using two-roll mills from Buzuluk (Komárov, Czech Republic). The composition of the tested compounds is presented in Table 2. Tested compounds were formed in sheets with a 2 mm thickness and then cured in an electrically heated press at 150 °C under a pressure of 4.9 MPa for the optimum vulcanization time determined according to the ISO 3417 standard.

### 2.4. Measurements

The thermal analysis of GTR and reclaimed GTR was performed using the simultaneous TGA/DSC model Q600 from TA Instruments (New Castle, DE, USA). Samples of reclaimed GTR weighing approximately 10 mg were placed in a corundum dish. The study was conducted in an inert gas atmosphere containing nitrogen (flow rate of 100 mL/min) in the range from 25 to 800 °C with a heating ramp of 20 °C/min. In order to better understand the thermal stability of GTR during extrusion, the TGA measurement in the air atmosphere in the same conditions was also performed. Volatile products that formed during thermal degradation of studied samples were also evaluated using Fourier-transform infrared spectroscopy (FTIR). During the TGA/DSC measurements, the volatile degradation products were directed, using a heated transfer line at 220 °C, to a Nicolet iS10 spectrometer from Thermo Scientific (Waltham, MA, USA). That setup allowed “on-line” characterization of volatile products released during TGA/DSC measurements. The timing offset of FTIR spectra compared to the TGA curves was related to the volume of the thermogravimetric apparatus chamber.

The morphology of GTR and reclaimed GTR was characterized by a Hitachi S3400 scanning electron microscope (Tokyo, Japan). Before the analysis, samples were coated with a thin layer of gold. Qualitative and quantitative analysis of GTR and reclaimed GTR surface composition was performed using energy-dispersive X-ray spectroscopy.

The FTIR spectra were measured in the range of 4000–650 cm^−1^ with a Momentμm microscope attached to a Nicolet iS50 FT-IR spectrometer (Waltham, MA, USA) equipped with the Specac Quest single-reflection diamond attenuated total reflectance (ATR) accessory. Spectral analysis was controlled by the OMNIC software package ver 9.8.372.

Volatile organic compounds emitted from reclaimed GTR were determined using static headspace and gas chromatography-mass spectrometry (SHS-GC-MS). Measurements were performed using a Shimadzu GC2010 PLUS GC-MS (Kyoto, Japan) equipped with a split/splitless inlet. The GC-MS system was equipped with an AOC5000 Headspace Auto-Sampler (Shimadzu, Kyoto, Japan). During analysis, the vial was transported by the injection unit from the tray to the agitator. When the sample achieved equilibrium, the headspace sample of a 2.5 mL volume was drawn from the vial and injected into the GC injector. The sampled vial was then returned by the injection unit to the tray.

The curing process of reclaimed GTR samples was investigated at 150 °C using a Monsanto R100S (Columbia City, IN, USA) rheometer with an oscillating rotor according to ISO 3417. For a better understanding of the changes that occurred during the reclaiming process, samples without a curing system were also investigated with the rheometer. Cure rate index (CRI) values were calculated in accordance with Equation (1):

(1) $$\mathrm{CRI}=\frac{100}{t_{90}-t_{2}}$$

where: *t_90_*—optimum vulcanization time, min; *t_2_*—scorch time, min.

In order to determine the aging resistance of the studied vulcanizates at elevated temperatures, the R_300_ parameter was determined. R_300_ defines the percentage reversion degree after a period of 300 s calculated from the time of reaching maximum torque (M_max._). R_300_ was calculated in accordance with Equation (2):

(2) $$R_{300}=\frac{M_{max.}-M_{300s}}{M_{max.}}\times 100\%$$

where: M_max_—maximum torque; M_300s_—torque 300 s after maximum torque.

The tensile strength, elongation at break, and modulus at 100% of elongation (M_100_) were estimated in accordance with ISO 37. Tensile tests were performed on a Zwick Z020 machine (Ulm, Germany) at a constant speed of 500 mm/min. Direct extension measurements were conducted periodically using an extensometer with sensor arms. Shore hardness type A was estimated using a Zwick 3130 durometer (Ulm, Germany) in accordance with ISO 7619-1. The tensile strength, elongation at break, modulus at 100%, and hardness were estimated based on five repetitions. 

## 3. Results and Discussion

### 3.1. Thermal Stability of GTR at Different Atmospheres

**Figure 3:**
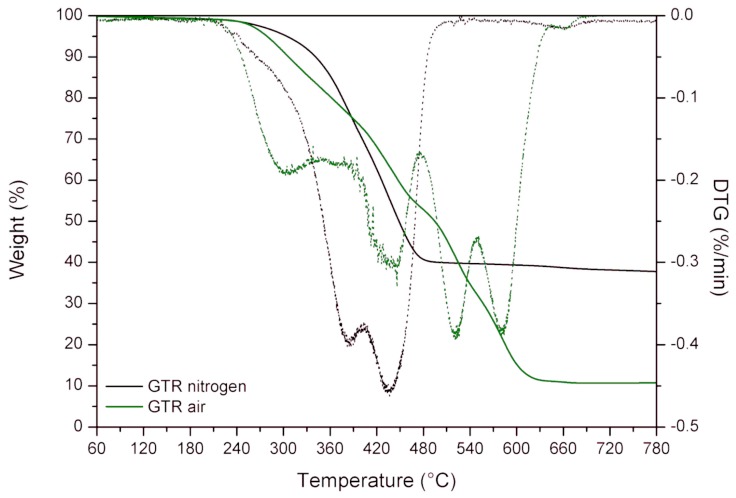
TGA and DTG curves for GTR in nitrogen and in air atmosphere.

In order to provide better insight into changes that occurred during the extrusion of GTR, the characteristics of the applied waste rubber were required. Thermogravimetric analysis (TGA) was applied to determine the thermal stability of GTR. The obtained results are presented in Figure 3 and summarized in Table 3. As could be observed in differential thermogravimetric (DTG) curves, two maxima were determined for GTR (384.5 and 435.7 °C, respectively) studied in the inert atmosphere of nitrogen and four maxima for GTR (302.7, 442.0, 521.1, and 581.1 °C, respectively) studied in air atmosphere. The first two peaks in DTG (regardless of the used atmosphere) corresponded to the temperatures of the maximum rate of thermal degradation of natural rubber (384.5 °C—in nitrogen and 302.7 °C—in air) and styrene-butadiene rubber (435.7 °C—in nitrogen and 442.0 °C—in air), which are the main elastomers used in car tire manufacturing [24]. The differences in the maximum rate of thermal degradation of natural rubber results from the thermooxidative degradation due to the presence of the oxygen. Two additional maxima (521.1 and 581.1 °C) for a study carried out in air are the result of carbon black oxidation [25], which corresponds to the char residue value presented in Table 3. Temperatures of T_-2%_, T_-5%_, T_-10%_, and T_-50%_ are related to the 2%, 5%, 10%, and 50% weight loss, respectively. As could be expected, the thermal stability of GTR was lower in air atmosphere, however, it should be pointed out that thermal decomposition of styrene-butadiene rubber was slower in the presence of air than in nitrogen. This is related to the secondary cross-linking of styrene-butadiene rubber affected by the high temperature in the presence of oxygen.

**Figure 4:**
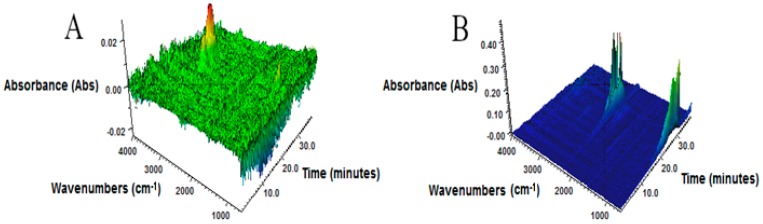
3D FTIR plots for volatile products emitted during the thermal decomposition of GTR: (**A**) nitrogen, (**B**) air atmosphere (heating ramp of 20 °C/min).

The 3D FTIR spectra determined for the volatile products emitted during the thermal decomposition of GTR in an inert atmosphere (A) and in air (B) are presented in Figure 4. In the case of the study carried out in nitrogen, a strong band in the range of 2900–2970 cm^−1^ appeared, which corresponds to the symmetric and asymmetric stretching vibrations of C-H bonds in the CH_2_ groups of volatile degradation products formed due to the degradation of hydrocarbons present in GTR. When the sample had access to the oxygen, absorbance maxima appeared at 2349 cm^−1^ and 876 cm^−1^, representing asymmetric stretching vibrations of CO_2_ [26] formed during thermo-oxidative degradation of the sample during thermogravimetric analysis.

### 3.2. Thermal Stability of Reclaimed GTR Collected from Different Zones of the Extruder Barrel

**Figure 5:**
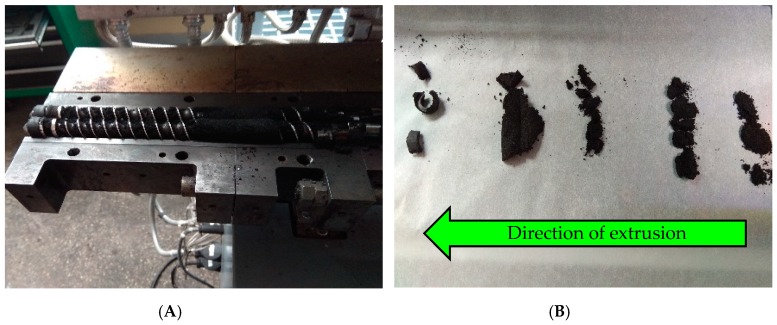
The appearance of: (**A**) open extruder barrel after stopping of reclaiming process (**B**) GTR collected from different zones of the extruder barrel.

In order to investigate the changes that occurred in the GTR structure during the reclaiming process, samples from different zones of the barrel were collected. In this case, samples after auto-thermal extrusion of ground tire rubber were evaluated. The reclaiming process was started and stabilized and after that, the gravimetric feeder and extruder were turned off. Subsequently, the extruder barrel divided in the plane was immediately opened using an electric screwdriver. The appearance of samples collected from different zones of the extruder barrel is shown in Figure 5.

The samples were collected directly from screws and examined using thermogravimetric analysis performed in a nitrogen atmosphere after at least 24 h of conditioning. Kleps et al. [27] confirmed that TGA as an analytical technique is a very useful tool to evaluate reclaimed rubbers and to establish the optimal parameters of the reclaiming/devulcanization process. The results of TGA measurements are presented in Table 4, showing the thermal properties of samples gathered from the same process and from three different barrel zones: Hopper, central, and last zone. As the high shear forces and higher temperature acted on GTR, the reclaiming process occurred, which is connected with the release of low molecular compounds from the complex structure of the waste tires. It was proven by the T_-2%_ value, which decreased with the progress of the reclaiming process (from 258.5 to 237.7 °C). The shift to a lower value shows either faster evaporation or decomposition of plasticizers or other low molecular additives present in GTR released during the process [28]. The GTR gathered from the last zone shows an increase in the T_-2%_ value (from 237.7 to 244.6 °C), which is the effect of partial evaporation/decomposition of low molecular compounds during the reclaiming process shifting decomposition temperatures to higher values. The mass loss in the 200–350 °C range did not change significantly with the process progression. The small increase was visible for the sample from the central zone in the range of 200–350 °C (from 11.0% to 13.9%) and the later drop of the value for GTR from the last zone (11.5%). This phenomenon corresponds to the aforementioned release of low molecular compounds and its evaporation/decomposition in a later stage. In the last zone, there is a material, which was influenced by factors favoring the reclaiming process (shear forces, temperature, and time). The scission of cross-links and degradation of main chains facilitated thermal decomposition of reclaimed GTR, which translated into higher values of mass loss in the range of 350–400 °C (hopper—17.6%, central zone—17.0%, last zone—20.4%) and 400–550 °C (hopper—30.8%, central zone—23.5%, last zone—41.2%), which are the temperatures of rubbers degradation. Those results show the partial reclaiming of GTR during the process, which was also confirmed by SEM-EDX described in Section 3.4.

### 3.3. Volatile Organic Compounds of Reclaimed GTR Collected from Different Zones of the Extruder Barrel

As the volatile organic compounds (VOCs) have a negative impact on the environment, further studies were focused on the estimation of the VOCs emitted during the reclaiming process. Sampling took place on the same basis as in the case of the thermal stability analysis (Section 3.2). The results of the SHS-GC-MS measurement are presented in Table 5. The applied measurement conditions allowed for the determination of seven compounds: Acetone (content in the range of: 2.2–5.5 mg/kg), methacrolein (0.9–2.1 mg/kg), 2-methylfuran (1.3–2.1 mg/kg), methyl vinyl ketone (1.6–3.9 mg/kg), methyl isobutyl ketone (8.3–25.9 mg/kg), cyclohexanone (2.4–9.5 mg/kg), and benzothiazole (6.5–40.6 mg/kg). Regardless of the sampling zone of the barrel, the highest concentration was detected for methyl isobutyl ketone and benzothiazole, which was also confirmed in our previous studies [6]. The first component is used for the synthesis of antiozonant 6PPD (a common component of tires) and the second one can be found as part of the structure of vulcanization accelerators. The presence of benzothiazole correlates with the presence of an unreacted curing system or scission of the sulfide cross-linking bonds present in GTR. Morand et al. [29] indicated that methacrolein and methyl vinyl ketone are formed during oxidation of polyisoprene, while other determined volatile compounds were also detected in natural rubber [30]. The total content of VOCs changed with the progress of the reclaiming process (hopper—23.2 mg/kg, central zone—89.6 mg/kg, last zone—56.8 mg/kg). The highest value was determined for reclaimed GTR from the central zone. The result proves previous assumptions about the release of low molecular compounds during the reclaiming process, which can be evaporated more easily when analyzed by the SHS-GC-MS method. Furthermore, the amount of VOCs for the reclaimed GTR from the last zone was lower than from the central zone, which indicates that part of the low molecular compounds evaporated into the environment during the process. It shows that the analysis of VOCs should be done in every step of GTR processing in order to evaluate changes in the structure of processed material and its influence on the environment.

### 3.4. SEM-EDX of Reclaimed GTR

**Figure 6:**
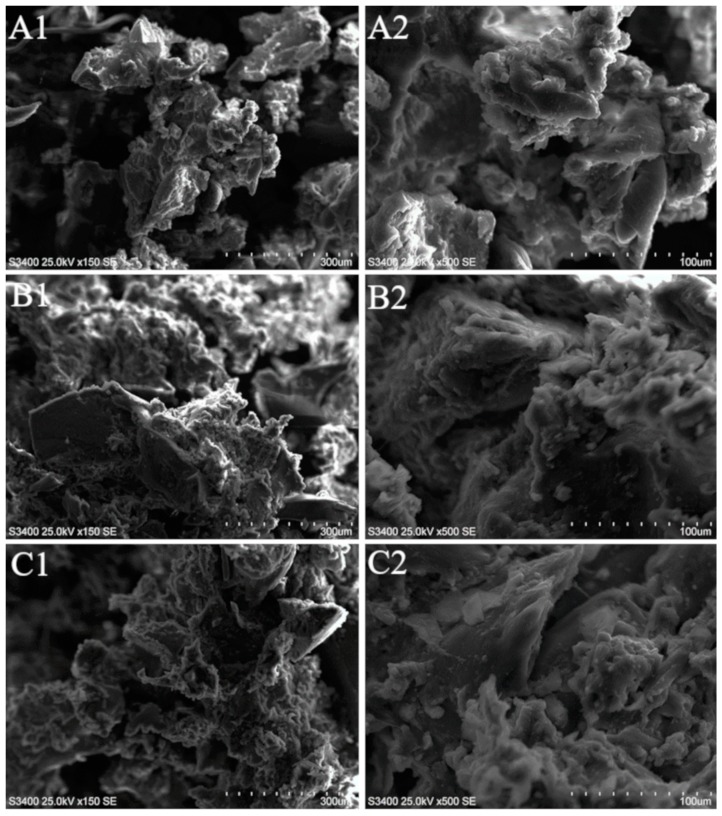
SEM images of: GTR (**A**1—magnification ×150, **A**2—magnification ×500), GTR-EXT (**B**1—magnification ×150, **B**2—magnification ×500), GTR-AUTO-EXT (**C**1—magnification ×150, **C**2—magnification ×500).

The SEM images presented in Figure 6 unraveled the surface morphology and homogeneity of untreated GTR, GTR-EXT, and GTR-AUTO-EXT. As displayed in the SEM micrographs, there is a minor difference between GTR and thermo-mechanically threated waste rubber. Due to the high shear forces and increased temperature, the reclaiming process occurred on the surface of waste rubber particles, resulting in its smoother surface and the formation of melt-like structures. It is revealed in the amount of white areas, which are increased in the SEM micrographs, especially in higher magnification, for GTR-EXT and GTR-AUTO-EXT compared to GTR. Moreover, the morphological structure of GTR-EXT and GTR-AUTO-EXT is very similar, indicating that heat generated during the process was enough to obtain the same devulcanization degree in both cases. 

The elemental composition of the studied samples is presented in Table 6. The results show that GTR and reclaimed GTR are mainly constituted of carbon (86.1–90.1 wt.%). Carbon is a principal element of rubber macromolecules as well as carbon black, which is commonly used as a reinforcement in tires. The presence of oxygen (1.8–5.1 wt.%) and silicon (1.2–3.3 wt.%) is directly related to the presence of silica, which is also used as a reinforcing additive; however, it may also be a result of simple contamination of waste tires by sand. The differences between the amount of detected oxygen and silicon in the tested samples are not a result of the reclaiming process but from the content of silica in GTR, which is in accordance with the oxygen to silicon ratio. Moreover, it might also be due to the presence of unreacted components commonly used in tire manufacturing, such as zinc oxide. The detection of sulfur (1.6–2.0 wt.%) corresponds to the applied vulcanization agents and accelerators. Even a small change in its content is significant due to the overall small amount of sulfur added during tire manufacturing (usually ~1 to 3 phr of sulfur). The decrease of the amount of the element indicates an ongoing reclaiming process, resulting in the scission of S–S and C–S cross-linking bonds and facilitating the evaporation of hydrogen sulfide and sulfide oxide to the atmosphere [10]. The zinc (2.5–3.2 wt.%) is used as a vulcanization activator. The samples were contaminated by iron (0.1–0.2 wt.%) and aluminum (0.1–2.6 wt.%). Both might derive from steel cord (aluminum used as deoxidizer) used in the construction of the tire.

### 3.5. FTIR Spectra of Reclaimed GTR

**Figure 7:**
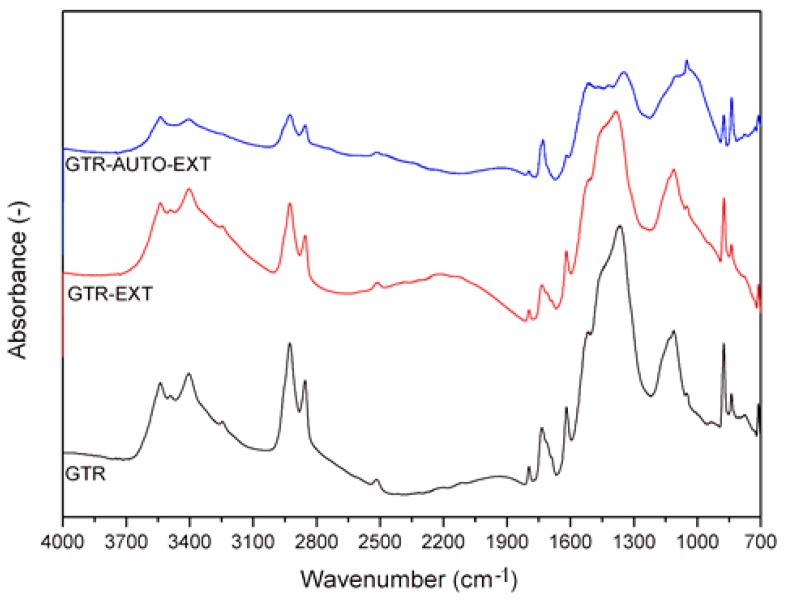
FTIR spectra of GTR, GTR-EXT, and GTR-AUTO-EXT samples.

The FTIR spectra obtained for GTR, GTR-EXT, and GTR-AUTO-EXT are presented in Figure 7. The results show no significant differences between the tested samples. It indicates that regardless of the applied reclaiming method, the chemical structure of the obtained reclaimed waste rubber did not change significantly compared to GTR. The absorbance maxima in the range of 3540–3410 cm^−1^ corresponds to O-H bonds vibrations, which suggest the presence of hydroxyl groups. The bands of C–H bonds of CH_2_ groups present in the aliphatic chains of elastomers are located in 2930 cm^−1^ and 2850 cm^−1^. The bands at approximately 1790 cm^−1^ and 1730 cm^−1^ are attributed to C=O bond vibrations, which occurred as a result of oxidative degradation. The band appears in every analyzed sample, which indicates that partial degradation took place during tire grinding. A characteristic peak for C=C bond present in benzene typical for styrene-butadiene rubber (common elastomer in the tire industry) is visible at approximately 1615 cm^−1^ and 670 cm^−1^. The characteristic band at approximately 1370 cm^−1^ corresponds to the C–H bonding of methyl groups, which confirms the presence of natural rubber in the examined rubber wastes. Another band for C–H vibrations is located at about 830 cm^−1^. In the range of 1100 cm^−1^ to 880 cm^−1^, C–O–C bonding as well as S=O, C–C, and C–O bonds are present in the chemical structure of the samples. The vibrations of C-S bonds can be observed at about 770 cm^−1^.

### 3.6. Curing Characteristics

**Figure 8:**
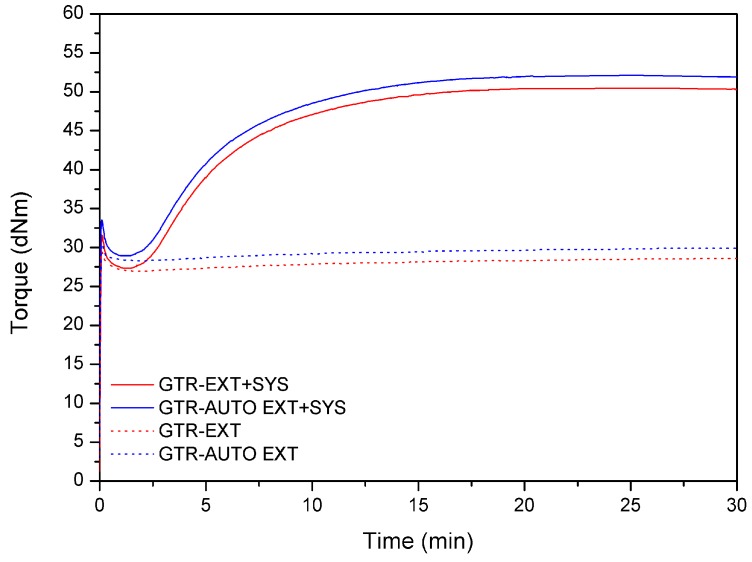
Curing curves for GTR-EXT and GTR-AUTO-EXT samples without and with a curing system (+SYS).

The curing curves of reclaimed GTR samples without (GTR-EXT and GTR-AUTO EXT) and with a curing system (GTR-EXT + SYS and GTR-AUTO EXT + SYS) are presented in Figure 8. As could be expected, the curing curves for GTR-EXT and GTR-AUTO EXT are almost linear, which indicates that revulcanization of reclaimed GTR without the curing system is rather limited. On the other hand, samples with a curing system (GTR-EXT + SYS and GTR-AUTO EXT + SYS) showed the typical course of curing curves.

The curing characteristics of GTR-EXT and GTR-AUTO-EXT with and without a sulfur curing system are presented in Table 7. The minimal torque values differ insignificantly depending on the applied reclaiming method (GTR-EXT and GTR-AUTO-EXT: 27.0 and 28.3 dNm, respectively). A similar change was observed for the samples including the curing system (coded as GTR-EXT + SYS and GTR-AUTO-EXT + SYS: 27.4 and 29.0 dNm, respectively). During the extrusion process, high shear forces and high temperatures act on GTR, enhancing the scission of cross-linking bonds and the degradation of polymer chains. This phenomenon causes the formation of low molecular compounds capable of partial secondary cross-linking of the reclaimed rubber structure during the process. This indicates that the auto-thermal method caused a more efficient partial cross-linking during the reclaiming process. The same dependence was observed for samples with the curing system. The minimal torque values of GTR-EXT are similar regardless of the presence of the curing system (27.0–27.4 dNm), and also for samples prepared with the auto-thermal method (28.3–29.0 dNm). The small difference of minimal torque values (in terms of the presence of a curing system) might result from the complex composition of waste tires.

In the case of GTR-EXT and GTR-AUTO-EXT, the torque values increased during the curing process for exactly 1.6 dNm even without the application of a curing system. It proves that the formation of low molecular compounds, capable of cross-linking, occurred during the reclaiming process via extrusion. However, the applied measurement methodology is not suitable for the determination of this phenomenon. The cross-linking process of reclaimed GTR is more efficient when a sulfur curing system is used. For both samples (GTR-EXT and GTR-AUTO-EXT) the torque increament value increased for 23.1 dNm. This shows that in the studied case, regardless of the heating conditions, the curing efficiency of the obtained reclaimed rubbers is the same. 

For GTR-EXT + SYS and GTR-AUTO-EXT + SYS samples, the scorch time was 2.6 and 2.5 min, the optimum cure time was 11.6 and 11.8 min, the cure rate index was 11.1 and 10.7 min^−1^, and the thermal aging resistance was 0.4% and 0.3 %, respectively. Those minimal changes in the mentioned values also indicate the fact that in the studied conditions, the heating method during extrusion of GTR did not affect its further curing.

### 3.7. Physico-Mechanical Properties

The physico-mechanical properties of the reclaimed rubbers are presented in Table 8. It was observed that for GTR-EXT and GTR-AUTO-EXT samples, the tensile strength (2.7 ± 0.1 MPa) and modulus 100 (1.5 MPa) did not change. A similar phenomenon was observed for the elongation at break values (205 ± 15% and 203 ± 7%, respectively), hardness (47 ± 1 and 51 ± 1 Sh A, respectively), and density (1.16 ± 0.01 and 1.16 ± 0.01 g/cm^3^, respectively). Small changes, or their lack, of the above mentioned properties show that the heating method during the reclaiming process had no influence on the physico-mechanical properties of the GTR-EXT and GTR-AUTO-EXT samples. 

The physico-mechanical properties of reclaimed GTR were enhanced by the addition of a sulfur curing system. The tensile strength of GTR-EXT + SYS and GTR-AUTO-EXT + SYS samples was 5.1 ± 0.3 and 4.6 ± 0.1 MPa, respectively. The difference between the tensile strength values of those samples is negligible. A similar tendency was observed when the rest of the parameters were compared. The elongation at break (201 ± 11 and 196 ± 7%, respectively), modulus at 100% (2.3 and 2.4 MPa, respectively), hardness (60 ± 1 and 59 ± 1 Sh A, respectively), and density (1.20 ± 0.01 and 1.19 ± 0.01 g/cm^3^, respectively) did not change significantly for GTR reclaimed by auto-thermal extrusion. 

In order to evaluate the physico-mechanical properties of laboratory manufactured reclaimed GTR, the obtained products were compared with commercially available reclaimed rubbers vulcanized in the same conditions as the GTR-EXT and GTR-AUTO-EXT samples. A comparison of the results is presented in Table 9. The results show that, excluding RO-1-S, the tensile strength presents a variation of 4.6 to 5.2 MPa, and elongation at break at 184% to 207%. The high values of the tensile strength (11.0 MPa) and elongation at break (419%) of RO-1-S probably result from the fact that the material used for the reclaiming process did not come from waste tires. 

## 4. Conclusions

The GTR reclaiming process is an important industrial method of rubber recycling, which is highly influenced by temperature. The use of lower temperatures may result in energy savings, which directly affects the reduction of the process costs. On the other hand, high shear forces applied to the cross-linked rubber particles causes their mutual friction and enhances exothermic reactions during reclaiming. As a consequence, a self-heating phenomenon during GTR reclaiming can be observed. The presented preliminary results showed that auto-thermal extrusion of GTR (process carried out without external heating) allows the production of reclaimed rubber with performance properties that are competitive with commercially available products. Moreover, the expected savings of energy and reduced emission of VOCs are also advantages of this method. Further research in this field should be focused on the improvement of processing at low temperatures (e.g. by plasticizers or other chemical additives) in order to ensure easier scale-up of this technology and deep analysis of processing costs. 

## Figures and Tables

**Table 1:** Extrusion conditions of ground tire rubber (GTR).

Extrusion Parameters	Sample Code	
	GTR-EXT	GTR-AUTO EXT
Material	GTR 0.4	GTR 0.4
throughput (kg/h)	2.5	2.5
Screws peed (rpm)	250	250
Set barrel temperature (°C)	60	Auto-thermal *
Barrel temperature after process stabilization (from hopper to die) (°C)	32/60/65/65/66/65/66/69/70/85/86	25/39/54/64/69/69/68/70/70/85/96
Pressure on extruder die (bar)	56.7	59.7
Screw torque (Nm)	15.1	15.5
Observations	Frayed GTR ribbon came out from the extrusion die, the material has a temperature over 120 °C. The emission of gases from extruded GTR was observed at the end of the extrusion die.	In auto-thermal conditions, the zones maintained the pre-set temperature. Like in the case of sample GTR-EXT, the emission of gases was observed during processing.

* extruder barrel heating/cooling system turned off.

**Table 2:** Rubber composition for testing reclaimed rubber.

Component	Recipe (phr)
Reclaimed rubber	100
Zinc oxide	2.5
Stearic acid	1.0
N-tert-butyl-2-benzothiazole sulfenamide (TBBS)	0.35
Sulfur	1.5

**Table 3:** Thermal decomposition characteristics of GTR in an inert (nitrogen) and in air atmosphere.

Sample Name	Decomposition Temperature (°C)				Maxima from DTG (°C)				Char Residue at 750 °C (%)
	T_-2%_	T_-5%_	T_-10%_	T_-50%_	T_max1_	T_max2_	T_max3_	T_max4_	
GTR nitrogen	258.5	304.3	341.3	448.4	384.5	437.5	-	-	38.0
GTR air	253.0	278.8	306.5	494.6	302.7	442.0	521.1	581.1	10.7

**Table 4:** Mass loss (Δm) as a function of the temperature range (ΔT) and T_-2%_ from TGA curves for reclaimed GTR collected from different zones of the extruder barrel.

Sample—Barrel Zone	ΔT (°C)	Δm (%)	T_-2%_ (°C)
GTR—hopper	200–350	11.0	258.5
	350–400	17.6	
	400–550	30.8	
reclaimed GTR—central zone	200–350	13.9	237.7
	350–400	17.0	
	400–550	23.5	
reclaimed GTR—last zone	200–350	11.5	244.6
	350–400	20.4	
	400–550	41.2	

**Table 5:** Volatile organic compounds determined using the SHS-GC-MS method.

Compound (mg/kg of Sample)	Sample—Barrel Zone		
	GTR—Hopper	Reclaimed GTR—Central Zone	Reclaimed GTR—Last Zone
Acetone	2.2	5.5	3.8
Methacrolein	0.9	2.1	1.7
2-methylfuran	1.3	2.1	1.9
Methyl vinyl ketone	1.6	3.9	2.8
Methyl isobutyl ketone	8.3	25.9	16.9
Cyclohexanone	2.4	9.5	4.9
Benzothiazole	6.5	40.6	24.8
Total content	23.2	89.6	56.8

**Table 6:** Elemental composition of GTR and reclaimed GTR determined by energy-dispersive X-ray spectroscopy.

Element (wt.%)	Sample		
	GTR	GTR-EXT	GTR-AUTO-EXT
Carbon	86.1	90.1	89.6
Oxygen	5.1	1.8	3.7
Silicon	3.3	1.2	2.0
Sulfur	2.0	1.6	1.8
Zinc	3.2	2.5	2.7
Iron	0.2	0.1	0.1
Aluminium	0.1	2.6	0.1

**Table 7:** Curing characteristics of GTR-EXT and GTR-AUTO-EXT with and without a sulfur curing system.

Item	Sample Code			
	GTR-EXT	GTR-AUTO EXT	GTR-EXT + SYS *	GTR-AUTO EXT + SYS
Minimal torque M_min_ (dNm)	27.0	28.3	27.4	29.0
Maximal torque M_max_ (dNm)	28.6	29.9	50.5	52.1
Torque increment ∆M (dNm)	1.6	1.6	23.1	23.1
Scorch time t_1_ (min)	-	-	2.6	2.5
Optimum cure time t_90_ (min)	-	-	11.6	11.8
Cure rate index CRI (min^−1^)	-	-	11.1	10.7
Thermal aging resistance R_300_ (%)	-	-	0.4	0.3

* sample contains a sulfur curing system presented in Table 2.

**Table 8:** Physico-mechanical properties of GTR-EXT and GTR-AUTO-EXT with and without a sulfur curing system.

Properties	Standard	Sample Code			
		GTR-EXT	GTR-AUTO EXT	GTR-EXT + SYS *	GTR-AUTO EXT + SYS *
Tensile strength (MPa)	ISO 37	2.7 ± 0.1	2.7 ± 0.1	5.1 ± 0.3	4.6 ± 0.1
Elongation at break (%)	ISO 37	205 ± 15	203 ± 7	201 ± 11	196 ± 7
Modulus at 100% (MPa)	ISO 37	1.5	1.5	2.3	2.4
Hardness (Sh A)	ISO 7619-1	47 ± 1	50 ± 1	60 ± 1	59 ± 1
Density at 25 °C (g/cm^3^)	ISO 1183	1.16 ± 0.01	1.16 ± 0.01	1.20 ± 0.01	1.19 ± 0.01

* sample contains a sulfur curing system presented in Table 2.

**Table 9:** A comparison between commercial reclaimed rubbers and reclaimed GTR produced in the laboratory scale (throughput: 2.5 kg/h).

Sample Code *	Supplier	Origin	Physico-Mechanical Properties			
			Tensile Strength (MPa)	Elongation at Break (%)	Hardness (°Sh A)	Density at 25 °C (g/cm^3^)
GTR-EXT	Laboratory manufacturing	Poland	5.1 ± 0.3	201 ± 11	60 ± 1	1.20 ± 0.01
GTR-AUTO-EXT	Laboratory manufacturing	Poland	4.6 ± 0.1	196 ± 7	59 ± 1	1.19 ± 0.01
RO-1-S	Geyer&Hosaja	Poland	11.0 ± 1.1	419 ± 11	53 ± 1	1.10 ± 0.05
RSZT	Chemical Worldwide Business	Russia	4.6 ± 0.2	207 ± 13	45 ± 1	1.17 ± 0.02
B-66/TS5	Chemical Worldwide Business	Netherlands	5.2 ± 0.5	184 ± 11	56 ± 1	1.22 ± 0.01

* All studied samples were compounded with a sulfur curing system and vulcanized in the same conditions.
